# Supplementary material for: Wine’s Phenolic Compounds and Health: A Pythagorean View
Source: Molecules. 2020 Sep 8;25(18):4105. doi: 10.3390/molecules25184105 (PMC7570485; doi:10.3390/molecules25184105)
Supplement: Supplementary file 1 [file molecules-25-04105-s001.pdf]

Table S1. Human long-term studies with dealcoholized red wine.

Subjects	Study Design	Subjects' Characteristics	Controls of Non-Healthy Subjects	Intervention Period	Run-in Period	Washout Between Interventions	Diet and Lifestyle	Intervention	Plasma Lipid Metabolism Variables	Plasma Glucose Metabolism Variables	Haemostatic System Variables	Plasma Inflammatory and Other Variables	Blood Pressure and Heart Rate Variables	Gut Microbiota and Fecal Variables	Urinary Variables	Ref
24:<br>19 M<br>5 W<br>40–70y	Randomized crossover	T2D, nonsmokers, regular drinkers, participants taking antihypertensive or lipid-lowering medication were not excluded	22 M, 38–62y, healthy, nonsmokers, regular drinkers	4 weeks each intervention	4 weeks of usual alcohol intake	No	No other alcohol	1. RW (women 230 mL/day, alcohol ~24 g/day, men 300 mL/day ~31 g/day)<br>2. DRW (equivalent volumes)<br>3. Water (equivalent volumes)	-	-	-	SPMs:<br>18-HEPE, RvE1-2, 18R-RvE3, 17-HDHA, RvD1, 17R-RvD1, RvD2, PD1, 14-HDHA, MaR-1<br>(Plasma SPMs were measured in 23 participants)<br>F2-isoprostanes	-	-	-	[84]
RESULTS												At BL:<br>T2D group vs. healthy control group:<br>↑ hs-CRP<br>↑ 18-HEPE<br>↑ 17-HDHA<br>↑ RvD1<br>↑ 17R-RvD1<br>Interventions:<br>NS				
22 M<br>20–65y	Open-label randomized crossover	Healthy, regular drinkers, nonsmokers, no medication	-	4 weeks each intervention	2 weeks no alcohol	No	Usual, no other alcohol	1. RW 375 mL/day, alcohol 41 g/day, polyphenols 2378 mg/L<br>2. DRW 375 mL/day, polyphenols 2284 mg/L<br>3. Water 375 mL/day	TG, TC, LDL, HDL	-	-	CYP450 eicosanoids:<br>20-HETE, total EETs, total DHETs<br>F2-isoprostanes<br>hs-CRP<br>SPMs:<br>18R/S-HEPE, RvE1-2-3, 18R-RvE3, 17S-HDHA, RvD1, RvD2, 17R-RvD1, 10S,17S-DHDHA, 14R/SHDHA, MaR-1, PD1<br>GGT	SBP (24 h, awake, asleep)<br>DBP (24 h, awake, asleep)<br>HR (24 h, awake, asleep)	-	20-HETE<br>F2-isoprostanes	[85]
RESULTS									RW vs. DRW, water:<br>↑ HDL			RW vs. DRW:<br>↑ 20-HETE<br>RW vs. DRW, water:<br>↑ F2-isoprostanes<br>↑ 18-HEPE<br>↑ RvD1<br>↑ 17R-RvD1<br>↑ GGT	RW vs. DRW, water:<br>↑ 24 h BP<br>↑ Awake SBP<br>↑ Awake DBP<br>RW vs. DRW:<br>↑ 24 h HR<br>↑ Awake HR<br>↑ Asleep HR<br>DRW vs. water:<br>↓ 24 h HR<br>↓ Awake HR<br>↓ Asleep HR		RW vs. DRW, water:<br>↑ 20-HETE<br>Urinary 20-HETE was positively related to plasma 18-HEPE only after RW	
24:<br>19 M<br>5 W<br>46–66y	Randomized crossover	T2D, nonsmokers, regular drinkers, participants taking antihypertensive or lipid-lowering medication were not excluded	-	4 weeks each intervention	4 weeks of usual alcohol intake	No	Usual, no other alcohol	1. RW (230 mL/day, alcohol 24 g/day W, 300 mL/day 31 g/day M)<br>2. DRW (equivalent volumes)<br>3. Water (equivalent volumes)	TG, TC, HDL, LDL, TC/HDL	Glucose<br>Insulin<br>HOMA-IR	Fibrinogen	hs-CRP<br>Homocysteine<br>Uric acid	SBP (24 h, awake, asleep)<br>DBP (24 h, awake, asleep)<br>HR (24 h, awake, asleep)	-	Na, Na/Cr	[86]
RESULTS									RW vs. water:<br>↑ TC<br>↑ TC/HDL	NS	NS	NS	RW vs. water:<br>↑ Awake SBP<br>↑ Awake DBP<br>RW vs. DRW:<br>↓ Asleep DBP<br>RW vs. DRW, water:<br>↑ 24 h HR<br>↑ Awake HR<br>↑ Asleep HR		NS<br>(A lower 24-h urinary sodium excretion during the RW period was no longer significant after correction for creatinine excretion)	
10 M<br>45–50y	Randomized crossover	Metabolic syndrome	10 healthy men	30 days each intervention	2 weeks no RW	15 days	Usual, no other alcohol	1. RW 272 mL/day<br>2. DRW 272 mL/day	TG, TC, HDL, LDL	Glucose	-	CRP<br>LPS<br>Uric acid<br>GGT, GOT, GPT	SBP, DBP	**Proteobacteria**<br>*Escherichia coli*<br>*Enterobacter cloacae*<br>**Fusobacteria**<br>**Actinobacteria**<br>*Bifidobacterium*<br>*Eggerthella lenta*<br>**Bacteroidetes**<br>*Bacteroides*<br>*Bacteroides uniformis*<br>*Parabacteroides distasonis*<br>*Prevotella*<br>**Firmicutes**<br>*Blautia coccoides–Eubacterium rectale* group<br>*Enterococcus*<br>*Clostridium* cluster IV<br>*Clostridium histolyticum* group<br>*Lactobacillus*<br>*Faecalibacterium prausnitzii*<br>*Roseburia*	-	[87]
RESULTS									At BL, metabolic syndrome group vs. healthy control group:<br>↑ TC<br>↑ TG<br>↓ HDL<br>RW, DRW vs. BL in metabolic	At BL, metabolic syndrome group vs. healthy control group:<br>↑ Glucose<br>RW, DRW vs. BL in metabolic syndrome group:		At BL, metabolic syndrome group vs. healthy control group:<br>↑ GGT<br>↑ CRP<br>↑ LPS<br>RW, DRW vs. BL in metabolic syndrome group:<br>↓ CRP<br>↓ LPS	At BL, metabolic syndrome group vs. healthy control group:<br>↑ SBP<br>↑ DBP	RW, DRW vs. BL in metabolic syndrome group:<br>**Proteobacteria**<br>↓ *Escherichia coli*<br>↓ *Enterobacter cloacae*<br>↑ **Fusobacteria**<br>**Actinobacteria**<br>↑ *Bifidobacterium*<br>↑ *Eggerthella lenta*		

									syndrome group:<br>↓ TG<br>↓ TC<br>↑ HDL<br>RW, DRW vs. BL in healthy control group:<br>↓ TC<br>After interventions, metabolic syndrome group vs. healthy control group:<br>↑ TG	↓ Glucose		RW, DRW vs. BL in healthy control group:<br>↓ GPT<br>After both interventions, metabolic syndrome group vs. healthy control group:<br>↑ GGT	RW, DRW vs. BL in metabolic syndrome group:<br>↓ SBP<br>↓ DBP	↑ **Bacteroidetes**<br>↓ *Bacteroides*<br>↑ *Prevotella*<br>↓ **Firmicutes**<br>↑ *Blautia coccoides–Eubacterium rectale* group<br>↓ *Clostridium* cluster IV<br>↓ *Clostridium histolyticum* group<br>↑ *Lactobacillus*<br>↑ *Faecalibacterium prausnitzii*<br>↑ *Roseburia*<br>RW, DRW vs. BL in healthy control group:<br>**Actinobacteria**<br>↑ *Bifidobacterium*<br>↑ *Eggerthella lenta*<br>↑ **Bacteroidetes**<br>↓ *Bacteroides uniformis*<br>**Firmicutes**<br>↑ *Faecalibacterium prausnitzii*<br>↑ *Roseburia*<br>The significantly lower number of *Bifidobacterium, Eggerthella lenta, Prevotella, Blautia coccoides–Eubacterium rectale* group, *Lactobacillus, Faecalibacterium prausnitzii and Roseburia* and a significantly higher number of *Proteobacteria, Firmicutes, Escherichia coli, Enterobacter cloacae, Bacteroides, Parabacteroides distasonis, Clostridium spp. and Clostridium histolitycum* observed at BL in the metabolic syndrome group vs. the healthy group, disappeared after the two red wine intervention periods<br>In the metabolic syndrome group:<br>The increase in *Actinobacteria* and *Lactobacillus* and the decrease in *Clostridium histolyticum* and *Escherichia coli* predicted the triglyceride reduction.<br>The increase in *Bifidobacterium* predicted cholesterol reduction.<br>The increase in *Faecalibacterium prausnitzii* predicted glucose reduction.<br>The decrease in *Clostridium* predicted the decrease in CRP.<br>The increase in *Bifidobacterium* and the decrease in *Enterobacter cloacae* predicted the reduction in plasma LPS.<br>In the healthy group:<br>The decrease in *Clostridium* predicted the decrease in SBP		
24 W<br>24–49y	Randomized crossover	Healthy, nonsmokers, regular drinkers, premenopausal	-	4 weeks each intervention	4 weeks of usual alcohol intake	No	Usual, no other alcohol	1. High volume RW (200 mL/day for lower-level alcohol consumers, 300 mL/day for higher-level alcohol consumers)<br>2. Low volume RW (100 mL/day for 4 days/week for lower-level alcohol consumers, 100 mL/day for higher-level alcohol consumers)<br>3. DRW (same volumes as high volume RW)	TG, TC, HDL, LDL	Glucose<br>Insulin	Fibrinogen	-	SBP (24 h, awake, asleep)<br>DBP (24 h, awake, asleep)<br>HR (24 h, awake, asleep)	-	24 h Na	[88]
RESULTS									High volume RW vs. DRW:<br>↑ HDL	NS	High volume RW vs. DRW:<br>↓ Fibrinogen		High volume RW vs. low volume RW, DRW:<br>↑ 24 h SBP<br>↑ Awake SBP<br>↑ 24 h DBP<br>High volume RW vs. low volume RW:<br>↑ Awake DBP		NS	
9 M<br>45–50y	Open-label randomized crossover	Healthy	-	20 days each intervention	No alcohol intake	No	Usual, no other alcohol	1. RW 272 mL/day, alcohol 30 g/day<br>2. DRW 272 mL/day<br>3. Gin 100 mL/day, alcohol 30 g/day	-	-	-	-	-	*Bifidobacterium*<br>*Enterococcus*<br>*Eggerthella lenta*	Hydroxybenzoic acids, 2,4-dihydroxybenzoic acid, 2,6-dihydroxybenzoic acid, 2,5-dihydroxybenzoic acid, 3,5-dihydroxybenzoic acid, Protocatechuic acid, Vanillic acid, Syringic acid, 4-hydroxybenzoic acid, 3-hydroxybenzoic acid, 4-hydroxyhippuric acid, 3-hydroxyhippuric acid, Gallic acid, 4-O-methyl gallic acid, Methyl gallic acid,	[89]

															Methyl gallic sulfate, Ethylgallate, Ethylgallate sulfate, Ethylgallate glucuronide 1,2, Hydroxyphenylacetic acids, Phenylacetic acid, 3-hydroxyphenylacetic acid, 2-hydroxyphenylacetic acid, 3,4-dihydroxyphenylacetic acid, Homovanillic acid, Hydroxycinnamic acids, m-coumaric acid, o-coumaric acid, p-coumaric acid, Caffeic acid, Ferulic acid, Sinapic acid, Hydroxyphenylpropionic acids, 3-(4-hydroxyphenyl)propionic acid, 3-(3-hydroxyphenyl)propionic acid, Dihydrocaffeic acid, Flavan-3-ols, (Epi)catechin glucuronide 1,2,3,4, (Epi)catechin sulfate 1,2,3, Methyl (epi)catechin glucuronide 1,2,3, Methyl (epi)catechin sulfate 1,2,3, Glycinates, Vanilloylglycine, Feruloylglycine, Hydroxyphenylvalerolactones, DHPV 1, DHPV 2, DHPV glucuronide 1,2, DHPV sulfate 1,2, MHPV 1, MHPV glucuronide 1, MHPV sulfate 1,2, Enterolactone, Pyrogallol	
RESULTS														RW, DRW vs. gin, BL:<br>↑ *Bifidobacterium*<br>↑ *Enterococcus*<br>↑ *Eggerthella lenta*	The increase in Bifidobacteria was significantly correlated with a higher excretion of syringic, p-coumaric, 4-hydroxybenzoic and homovanillic acids.<br><br>68.5% of changes in Bifidobacteria can be predicted by syringic acid and 4-hydroxybenzoic acid changes.<br><br>Significant inverse associations were found between changes in *Eggerthella lenta* tertiles and changes in hydroxycinnamic acid concentrations and between changes in *Enterococcus* tertiles and changes in 3,4-dihydroxyphenylacetic acid concentration.	
8<br>45–50y	Randomized crossover	Healthy	-	20 days each intervention	2 weeks no alcohol	No	Usual, no other alcohol	1. DRW 272 mL/day<br>2. RW 272 mL/day<br>3. Gin 100 mL/day	-	-	-	-	-	benzoic acids<br>- gallic acid<br>- 3,5-dihydroxybenzoic acid<br>- protocatechuic acid<br>- 3-O-methylgallic acid<br>- 4-hydroxybenzoic acid<br>- 3-hydroxybenzoic acid<br>- vanillic acid<br>- syringic acid<br>- benzoic acid<br>- salicylic acid<br>phenylacetic acids<br>- 4-hydroxyphenylacetic acid<br>- 3-hydroxyphenylacetic acid<br>- phenylacetic acid<br>phenylpropionic acids<br>- 3-(3,4-dihydroxyphenyl)propionic acid<br>- 3-(3-hydroxyphenyl)propionic acid<br>- phenylpropionic acid<br><br>valeric acids<br>- 4-hydroxy-5-(3′,4′-dihydroxyphenyl)valeric acid<br>- 4-hydroxy-5-(3′-hydroxyphenyl)valeric acid<br>- 4-hydroxy-5-(phenyl)valeric acid<br>cinnamic acids<br>- caffeic acid<br>- p-coumaric acid<br>- ferulic acid	-	[90]
RESULTS														RW, DRW vs. BL:<br>↑ 3,5-dihydroxybenzoic acid<br>↑ 3-O-methylgallic acid<br>↑ vanillic acid<br>↑ syringic acid<br>↑ phenylpropionic acid<br>↑ 4-hydroxy-5-(phenyl)valeric acid<br>↑ p-coumaric acid<br>RW vs. BL:<br>↑ protocatechuic acid		
10 M<br>45–50y	Randomized crossover	Healthy, nonsmokers, moderate alcohol consumers	-	20 days each intervention	15 days no alcohol or RW	No	Usual, no other alcohol	1. RW 272 mL/day<br>2. DRW 272 mL/day<br>3. Gin 100 mL/day	-	-	-	LPS<br>LBP	-	**Proteobacteria**<br>*Escherichia coli*<br>**Actinobacteria**<br>*Bifidobacterium*<br>**Bacteroidetes**<br>*Bacteroides*<br>*Parabacteroides distasonis*<br>*Prevotella*<br>**Firmicutes**<br>*Blautia coccoides–Eubacterium rectale* group<br>*Enterococcus*	-	[80]

														Clostridium cluster IV<br>*Clostridium histolyticum* group<br>*Lactobacillus*		
RESULTS												The change in LPS concentration after each intervention period correlated positively with the HOMA-IR, significantly so after the DRW intervention<br>LPS concentrations correlated negatively with *Prevotella* and *Bifidobacterium*		RW vs. DRW, gin:<br>↑ *Bacteroidetes*<br>↑ *Firmicutes*<br>↑ *Prevotella*<br>Gin vs. RW, DRW:<br>↓ *Bacteroidetes*<br>↓ *Firmicutes*<br>RW, DRW vs. gin:<br>↑ *Bifidobacterium*		
73 M (-6)<br>55–75y	Open-label randomized crossover	High cardiovascular risk	-	4 weeks each intervention	2 weeks no alcohol	No	Usual, no other alcohol, no alcohol-free beer	1. RW 272 mL/day, alcohol 30 g/day, polyphenols 798 mg/day<br>2. DRW 272 mL/day, polyphenols 733 mg/day<br>3. Gin 100 mL/day, alcohol 30 g/day	TC, TG, LDL, HDL, LDL/HDL ratio, ApoA-I, ApoA-II, Lp(a), ApoB, ApoC-I, ApoC-III, GH, leptin, adiponectin	Glucose<br>Insulin<br>HOMA-IR	-	Homocysteine,<br>Vit. B12,<br>folic acid	-	-	-	[91]
RESULTS									RW, gin vs. BL, DRW:<br>↑ HDL<br>↑ ApoA-II<br>RW, gin vs. DRW:<br>↓ LDL/HDL ratio<br>↓ ApoA-I<br>RW vs. DRW, gin:<br>↓ Lp(a)<br>RW vs. BL:<br>↓ LDL<br>↓ LDL/HDL ratio<br>DRW vs. BL:<br>↓ ApoB	RW, DRW vs. BL, gin:<br>↓ Insulin<br>↓ HOMA-IR<br>(↓ HOMA-IR even after excluding the 15 diabetic subjects)		RW, DRW vs. BL, gin:<br>↓ Folic acid				
73 M (-6)<br>55–75y	Open-label randomized crossover	High cardiovascular risk, moderate alcohol consumers	-	4 weeks each intervention	2 weeks no alcohol	No	Usual, no other alcohol	1. RW 272 mL/day, alcohol 30 g/day, polyphenols 798 EGA/day<br>2. DRW 272 mL/day, polyphenols 733 EGA/day<br>3. Gin 100 mL/day, alcohol 30 g/day	-	-	-	NO	SBP, DBP, HR	-	-	[92]
RESULTS												DRW vs. BL, gin:<br>↑ NO	DRW vs. BL, gin:<br>↓ SBP<br>↓ DBP<br>Changes in BP correlated with changes in NO after the DRW period<br>Changes in SBP correlated with changes in NO after the RW intervention			
61 M<br>≥55 y	Randomized crossover	High cardiovascular risk	-	4 weeks each intervention	15 days no alcohol	No	Usual, no other alcohol	1. DRW 272 mL/day, alcohol 30 g/day<br>2. RW 272 mL/day 30 g/day<br>3. Gin 100 mL/day, alcohol 30 g/day	-	-	-	-	-	-	Acetate, Acetoacetate, Acetone, cis-Aconitate, Acetylcarnitine, Alanine, Betaine, Carnitine, Citrate, Creatine, Creatinine, Dimethylamine (DMA), Formate, Fucose, Glycine, Glycylproline, Glucose, Hippurate, Histidine, 2-Hydroxyisobutyrate, 3-Hydroxyisovalerate, 3-Hydroxymandelate, 4-Hydroxyphenylacetate, Indole-3-acetate, Lactate, Leucine, Lysine, Malonate, Mannitol, n-Methylhistidine, 1-Methylnicotinamide, Methylsuccinate, 3-Methyl-2-oxovalerate, N-N Dimethylglycine (DMG), Tartrate, N-Phenylacetylglycine (PAG), Succinate, Taurine, Trigonelline, Trimethylamine (TMA), Trimethylamine-N-oxide (TMAO), Threonine, Tyrosine, Urea, Valine	[93]
RESULTS															RW, DRW vs. BL, gin:<br>↑ Tartrate<br>↑ 3-methyl-2-oxovalerate<br>RW vs. BL, DRW, gin:<br>↑ Mannitol<br>DRW vs. BL, RW, gin:<br>↑ Hippurate<br>DRW vs. BL, gin:<br>↑ 4-hydroxyphenylacetate	
19 M	Randomized crossover	Healthy, nonsmokers, regular alcohol consumers	-	4 weeks each intervention	-	-	Usual, no other alcohol	1. RW 450 mL/day, alcohol 41.4 g/day<br>2. DRW 450 mL/day	TG, TC, HDL, LDL	Glucose<br>Insulin	Platelets	Plasma total antioxidant capacity (measured by the TEAC assay), NF-kB, Adiponectin, Creatinine, ALP, GOT, GPT, GGT	-	-	F2-isoprostane (8-iso $PGF_{2\alpha}$)	[94]
RESULTS									RW vs. DRW:<br>↑ HDL	NS	NS	RW vs. DRW:<br>↑ Adiponectin			RW vs. DRW:<br>↑ 8-iso-$PGF_{2\alpha}$	

												↓ GGT				
10 M<br>45–50y	Randomized crossover	Healthy	-	20 days each intervention	15 days no alcohol or RW	No	Usual, no other alcohol	1. DRW 272 mL/day, polyphenols 733mEqGA/day<br>2. RW 272 mL/day, polyphenols 798 mEqGA/day<br>3. Gin 100 mL/day	TC, TG, HDL, LDL	Glucose	-	CRP<br>Uric acid<br>GOT, GPT, GGT	DBP<br>SBP	**Proteobacteria**<br>**Fusobacteria**<br>**Actinobacteria**<br>*Bifidobacterium*<br>*Eggerthella lenta*<br>**Bacteroidetes**<br>*Bacteroides*<br>*Bacteroides uniformis*<br>*Prevotella*<br>***Firmicutes***<br>*Blautia coccoides–Eubacterium rectale group*<br>*Enterococcus*<br>*Clostridium*<br>*Clostridium histolyticum group*<br>*Lactobacillus*	-	[83]
RESULTS									RW, DRW, gin vs. BL:<br>↓ TG<br>RW, DRW vs. BL, gin:<br>↓ HDL<br>RW vs. BL, DRW, gin:<br>↓ TC	NS		RW, DRW vs. BL, gin:<br>↓ CRP<br>↓ GGT<br>↓ GOT<br>RW vs. BL, DRW, gin:<br>↓ Uric acid	RW vs. BL, DRW, gin:<br>↓ DBP<br>RW, DRW vs. BL, gin:<br>↓ SBP	↑ **Proteobacteria** *(RW* vs. *BL, gin)*<br>↑ **Fusobacteria** *(RW* vs. *BL, gin; DRW* vs. *BL)*<br>**Actinobacteria** *(NS)*<br>↑ *Bifidobacterium (RW, DRW* vs. *BL, gin)*<br>↑ *Eggerthella lenta (RW, DRW* vs. *BL, gin)*<br>↑ **Bacteroidetes** *(RW* vs. *BL, DRW, gin)*<br>↑ *Bacteroides (RW* vs. *BL, gin)*<br>↑ *Bacteroides uniformis (RW* vs. *BL, gin)*<br>↑ *Prevotella (RW* vs. *BL, gin;*<br>↓ *Prevotella gin* vs. *BL, RW, DRW)*<br>↑ ***Firmicutes*** *(RW* vs. *BL, DRW, gin)*<br>↑ *Blautia coccoides–Eubacterium rectale* group *(RW* vs. *BL, gin; DRW* vs. *BL)*<br>↑ *Enterococcus (RW, DRW* vs. *BL, gin)*<br>↑ *Clostridium (gin* vs. *BL, RW, DRW)*<br>↑ *Clostridium histolyticum* group *(gin* vs. *BL, RW, DRW)*<br>*Lactobacillus (NS)*<br>Significant univariate correlations:<br>*Bacteroides* with DBP, SBP, TG, TC, HDL<br>*Enterococcus* with SBP<br>*Bifidobacterium* with TC, CRP<br>*Lactobacillus* with HDL, CRP<br>Multivariate regression analysis:<br>↑ *Bacteroides* predicted ↓ DBP ↓ SBP ↓ TG ↓ HDL.		
8<br>25–40y	Randomized crossover	Healthy	-	1 week each intervention	2 days	1 month	Low phenolic diet, no alcohol	1. Low phenolic diet (LPD)<br>2. DRW + LPD	TC, TG, LDL, HDL	-	-	*Inflammatory marker:*<br>CRP<br>*Antioxidant enzymes:*<br>Superoxide dismutase, Catalase, Glutathione peroxidase, Glutathione reductase<br>*Endogenous antioxidants:*<br>Uric acid, Albumin, Bilirubin<br>*Antioxidant capacity:*<br>FRAP, ORAC, GSH/GSSG<br>*Hepatic profile:*<br>GOT, GPT, GGT	-	-	-	[95]
RESULTS									NS			LPD + DRW vs. BL, LPD:<br>↑ Glutathione reductase activity<br>↑ Catalase activity<br>↑ Superoxide dismutase activity<br>LPD vs. BL:<br>↓ Glutathione reductase activity<br>↓ Catalase activity<br>↓ Superoxide dismutase activity<br>DRW + LPD vs. BL:<br>↑ FRAP				
67 M<br>55–75y	Open-label randomized crossover	High cardiovascular risk, moderate alcohol consumers	-	4 weeks each intervention	2 weeks no alcohol	No	Usual, no other alcohol	1. RW 272 mL/day, alcohol 30 g/day<br>2. DRW 272 mL/day<br>3. Gin 100 mL/day, alcohol 30 g/day	-	-	-	*Inflammatory markers*:<br>CD40a, CD40L, CRP, E-selectin, ICAM-1, IL-1α, IL-10, IL-16, IL-18, IL-6, MCP-1, MCP-2, MCP-3, MDC, MIP-1α, MPIF1, TNF-α, VCAM-1<br>*Adhesion molecules on T-lymphocytes and monocytes*:<br>VLA-4, LFA-1, Mac-1, SLe^x, CD40, CD36, CCR2	-	-	-	[82]
RESULTS												*Inflammatory markers*:<br>DRW vs. RW, Gin:<br>↓ E-selectin<br>RW, DRW vs. Gin, BL:<br>↓ ICAM-1<br>↓ IL-6				

												RW, Gin vs. DRW, BL:<br>↑ IL-10<br>↓ MDC<br>RW, DRW, Gin vs. BL:<br>↓ CD40a<br>↓ CD40L<br>↓ IL-16<br>↓ MCP-1<br>↓ VCAM-1<br>*Adhesion molecules on T-lymphocytes and monocytes*:<br>Gin vs. RW, DRW:<br>↑ LFA-1 on TL<br>DRW vs. Gin:<br>↓ Mac-1 on monocytes<br>RW, DRW vs. Gin:<br>↓ CCR2 on monocytes<br>RW, DRW vs. BL:<br>↓ SLe^x on TL and monocytes<br>↓ Mac-1 on monocytes<br>↓ CCR2 on monocytes				
72 (-1)<br>36 M<br>36 W<br>22–56y	Open-label randomized crossover	Healthy, nonsmokers, moderate alcohol consumers	-	3 weeks each intervention	2 weeks	3 weeks	Usual, no other alcohol	Group 1 (12M, 12W):<br>1. RW 30 g/day for M, 20 g/day for W<br>2. DRW<br>Group 2 (12M, 12W):<br>1. Beer 30 g/day for M, 20 g/day for W<br>2. DB<br>Group 3 (12M, 12W):<br>1. Ethanol 30 g/day for M, 20 g/day for W<br>2. Water	-	-	-	Adiponectin	-	-	-	[96]
RESULTS												RW vs. BL in women:<br>↑ Adiponectin<br>Beer, ethanol vs. BL in men:<br>↑ Adiponectin				
78	Randomized	Healthy, nonsmokers	-	6 weeks	1 week, dietary restrictions as during the study	-	Coffee, green or black tea <300 mL/day, fruit juices <400 mL/day, no antioxidant vitamins enriched juices, grape juices, alcohol and probiotics	27 subjects:<br>RW 200 mL/day, 293 mg catechin equivalents<br>26 subjects:<br>DRW 175 mL/day, 272 mg catechin equivalents<br>25 controls:<br>No intervention	-	-	-	Apoptotic T cells, phagocytic activity of granulocytes and monocytes, respiratory burst of granulocytes and monocytes	-	-	-	[97]
RESULTS												RW, control vs. BL:<br>↓ Respiratory burst of monocytes				
29	-	T2D	23 healthy	28 days	-	-	Usual	Diabetics were given:<br>1. Muscadine wine (MW) n = 10,150 mL/day (subjects on MW were regular wine consumers)<br>2. Muscadine juice (MJ) n = 10 150mL/day<br>3. DW n = 9150 mL/day<br>Healthy subjects were given:<br>1. MJ n = 8150 mL/day<br>2. No intervention n = 15	TC, TG, LDL, HDL, TC/HDL, erythrocyte membrane fatty acids (SAT, MUFA, PUFA)	Glucose, insulin, glycated hemoglobin, glucose/insulin ratio	-	K, Na, Cl, CO2, ALP, Ca, P, Mg	SBP, DBP	-	-	[81]
RESULTS									MJ in healthy subjects vs. in T2D:<br>↓ TG<br>In T2D, MJ vs. BL:<br>↓ HDL	In T2D, DW vs. BL:<br>↓ Insulin		In T2D, MW vs. BL, MJ, DW; MW in T2D vs. no intervention group:<br>↓ Na<br>↓ Ca<br>In T2D, MW vs. MJ; MW in T2D vs. no intervention group:<br>↓ Cl<br>In T2D, MW vs. MJ, DW:<br>↓ ALP<br>In T2D, MJ vs. BL, MW; MJ in T2D vs. juice healthy group, no intervention group:<br>↑ P	NS			
36 M (-2)<br>35–70y	Randomized crossover	Healthy, nonsmokers, moderate alcohol consumers	-	4 weeks each intervention	-	No	Usual, no other alcohol	1. RW 450 mL/day, alcohol 40 g/day<br>2. DRW 450 mL/day	Ultrasound measurement of liver, abdominal, and subcutaneous fat depots, TC, TG, LDL, HDL	ISI (measured on 17 subjects)	-	Adiponectin<br>Resistin	-	-	-	[98]
RESULTS									Ultrasound NS<br>RW vs. DRW:<br>↑ HDL	NS		RW vs. DRW:<br>↑ Adiponectin<br>Changes in resistin correlated significantly with changes in ISI				

78 (-4)<br>18–50y	Randomized	Healthy, nonsmokers	25 healthy subjects were given water	6 weeks	1 week, dietary restrictions as during the study	-	Coffee, black or green tea <150 mL/day each, fruit juice <200 mL/day each, no grape juice, multivitamin juices or alcohol	1. RW n = 27 200 mL/day<br>2. DRW n = 26 175 mL/day<br>3. No intervention n = 25	-	-	-	TPP, TEAC, Vit. C, α-Tocopherol, Uric acid, Albumin, Bilirubin, Endogenous DNA strand breaks in peripheral leukocytes (TM_0), Exogenous DNA strand breaks in peripheral leukocytes (TM_{300})	-	-	-	[99]
RESULTS												RW vs. BL:<br>↑ TPP<br>↓ TM_0<br>No intervention vs. BL:<br>↑ Uric acid<br>DRW vs. BL:<br>↓ Bilirubin				
28 M (-4)<br>39–65y	Open-label randomized crossover	Healthy, nonsmokers, regular drinkers	-	4 weeks each intervention	2 weeks no alcohol	No	Usual diet, no alcohol, no antioxidant supplementation or over-the-counter medication, tea intake ≤2 cups/day	1. RW 375 mL/day 39g/day<br>2. DRW 375 mL/day<br>3. Beer 3 × 375 mL/day 41 g/day<br>4. Control (no alcohol or grape products intake)	-	-	-	GGT	BP (awake, asleep, 24 h)<br>HR (awake, asleep, 24 h)<br>FMD and GTNMD of brachial artery	-	ET-1	[100]
RESULTS												RW, beer vs. DRW, control:<br>↑ GGT	RW vs. control, DRW:<br>↑ 24 h SBP<br>↑ Awake SBP<br>↑ 24 h HR<br>↑ Asleep HR<br>Beer vs. control:<br>↑ 24 h SBP<br>↑ Awake SBP<br>Beer vs. control, DRW:<br>↑ 24 h HR<br>↑ Asleep HR<br>RW, beer vs. DRW:<br>↑ Awake HR		There was no specific effect of RW, DRW or beer on ET-1.<br><br>However, post hoc comparison of the averaged results from the 2 alcohol periods (beer and RW) and nonalcohol periods (control and DRW) found that alcohol increased urine ET-1 excretion	
24 M<br>30.6 ± 1.4y	Single-blind randomized crossover	Healthy, nonsmokers, moderate alcohol consumers	-	2 weeks each intervention	2 months no vitamin supplements or medication	1 week	Usual diet, no alcohol or food products rich in anthocyanins or polyphenols, no vitamin supplements or medication	1. RW 500 mL/day<br>2. DRW 500 mL/day<br>3. Red grape juice (RGJ) 500 mL/day<br>4. EtOH 500 mL/day	-	-	-	Lymphocyte-specific cytokines:<br>IL-2<br>IL-4<br>Monocyte-specific cytokines:<br>TGF*β*<br>TNF*α*<br>TNF*α*-mRNA<br>Phagocytic activity of granulocytes and monocytes,<br>Apoptotic activity of T-lymphocytes,<br>Lymphocyte proliferation,<br>NK cell lytic activity	-	-	-	[101]
RESULTS												NS				
18 M<br>25–71y	Randomized crossover	Healthy, smokers, light to moderate drinkers, no medication	-	2 weeks each intervention	1 week no alcohol	1 week	Usual, no other alcohol, no antioxidant supplements or over-the-counter medication	1. RW 375 mL/day, 1200 mg/L polyphenols<br>2. WW 375 mL/day, 345 mg/L polyphenols<br>3. DRW 500 mL/day, 905 mg/L polyphenols	TC, TG, HDL, LDL	-	-	F2-isoprostanes<br>Arachidonic acid<br>Uric acid<br>Vit. C<br>β-Carotene<br>α-Tocopherol<br>γ-Tocopherol<br>GGT	-	-	F2-isoprostanes	[102]
RESULTS									NS			DRW vs. RW, WW:<br>↓ F2-isoprostanes<br>RW, WW vs. DRW:<br>↑ Uric acid<br>↓ β-Carotene<br>RW, WW vs. BL:<br>↑ GGT<br>DRW vs. BL:<br>↓ GGT			DRW vs. RW:<br>↓ F2-isoprostanes	
6 M<br>22–29y<br><br>Two withdrew from the study 2 days before its completion. As a result, the sweat and blood data for the last metabolic period were not obtained	Randomized crossover	Healthy, moderate alcohol consumers	-	18 days each intervention	3 days	No	The subjects were housed in a closed, apartment-like metabolic unit for the duration of the experiment, except for occasional supervised walks, and were given	1. W 1l/day<br>2. DW 1l/day<br>3. EtOH 1l/day (9,3% alcohol)<br>4. Deionized water 1l/day<br>The daily beverage intake was given in divided doses over a 12 h period	-	-	-	Zn balance<br>P balance<br>Ca balance<br>Mg balance<br>Na balance<br>K balance<br>N balance<br>Fluid balance<br>BUN<br>Total protein<br>Creatinine<br>Uric acid	-	Zn, P, Ca, Mg, Na, K, N	Zn, P, Ca, Mg, Na, K,<br>Total N, creatinine, urea N, uric acid,<br>Urine osmolality<br>Urine output volume	[103–106]

for these subjects, and the summary of their urine and fecal data for this period represents 12 instead of 18 days.	a controlled diet. Daily exercise routine: two half-hour sessions of walking on a treadmill, one 15 min session on a bicycle ergometer and a 15 min exercise session of the individual's choice, decided upon before the study and maintained throughout.			
RESULTS		DW, W vs. EtOH, deionized water:<br>↑ Zn balance<br>EtOH vs. W, DW:<br>↓ Ca balance<br>↓ Mg balance<br>Deionized water vs. DW:<br>↓ Ca balance<br>DW vs. W, EtOH, deionized water:<br>↑ P balance<br>EtOH vs. W, DW, deionized water:<br>↓ K balance<br>W vs. EtOH:<br>↑ Fluid balance	W, DW vs. EtOH:<br>↓ Zn<br>DW vs. deionized water:<br>↓ Zn<br>DW vs. EtOH, deionized water:<br>↓ Ca<br>W vs. EtOH:<br>↓ Ca<br>↑ N ($p = 0.05$)<br>W, DW vs. EtOH, deionized water:<br>↓ P<br>↓ Mg	W, EtOH vs. DW, deionized water:<br>↑ Zn<br>↑ P<br>W vs. EtOH:<br>↑ P<br>W, EtOH vs. deionized water:<br>↑ Mg<br>DW vs. EtOH, deionized water:<br>↓ Na<br>EtOH vs. W, DW, deionized water:<br>↑ K<br>DW vs. W, EtOH, deionized water:<br>↓ Urine osmolality<br>Mean of alcohol periods vs. mean of non-alcohol periods:<br>↑ Total N<br>↑ Uric acid<br>↑ Urea N<br>Deionized water vs. DW:<br>↑ Urea N<br>There was a significant difference between alcohol (W and EtOH) and nonalcohol (DW and deionized water) periods for phosphorus (P).

M = men; W = women; y = years; T2D = type 2 diabetes; BMI = body mass index; RW = red wine; DRW = dealcoholized red wine; SMPs = specialised pro-resolving mediators of inflammation; 18-HEPE = 18-hydroxyeicosapentaenoic acid; RvE1-2, 18R-RvE3 = E-series resolvins; 17-HDHA = 17-hydroxydocosahexaenoic acid; RvD1, 17R-RvD1, RvD2 = D-series resolvins; PD1 = protectin D1; 14-HDHA = 14-hydroxydocosahexaenoic acid; MaR-1 = maresin-1; NS = non significant; TG = triglycerides; TC = total cholesterol; HDL = high density lipoproteins; LDL = low density lipoproteins; 20-HETE = 20-Hydroxyeicosatetraenoic acid; EETs = epoxyeicosatrienoic acids; DHETs = dihydroxyeicosatrienoic acids; hs-CRP = high sensitivity C-reactive protein; 18R/S-HEPE = 18R/S-hydroxy-eicosapentaenoic acid; 18R-RvE3 = 18R-resolvin E3; 17S-HDHA = 17S-hydroxydocosahexaenoic acid; 10S,17S-DHDHA = 10S,17S-dihydroxy-docosahexaenoic acid; 14R/SHDHA = 14-hydroxydocosahexaenoic acid; PD1 = protectin-1; BP = blood pressure; HR = heart rate; DBP = diastolic blood pressure; SBP = systolic blood pressure; HOMA-IR = homeostatic model assessment for insulin resistance; Na = sodium; Cr = creatinine; CRP = C-reactive protein; LPS = lipopolysaccharide; BL = baseline; LBP = lipopolysaccharide binding protein; ApoA-I = apolipoprotein A1; ApoA-II = apolipoprotein A2; Lp(a) = Lipoprotein(a); ApoB = apolipoprotein B; ApoC-I = apolipoprotein C1; ApoC-III = apolipoprotein C3; GH = growth hormone; EGA/day = Eq Gallic Acid/day; NO = nitric oxide; TEAC = trolox equivalent antioxidant capacity; NF-kB = nuclear factor kappa-light-chain-enhancer of activated B cells; 8-iso $PGF_{2\alpha}$ = 8-iso prostaglandin $F_{2\alpha}$; FRAP = ferric reducing ability; ORAC = oxygen radical absorbance capacity; GSH/GSSG = reduced glutathione/oxidized glutathione; CD40a = cluster of differentiation 40 antigen; CD40L = cluster of differentiation 40 ligand; ICAM-1 = intercellular adhesion molecule 1; IL = interleukin, MCP = monocyte chemotactic protein; MDC = macrophage-derived chemokine; MIP-1α = macrophage inflammatory protein 1α; MPIF1 = myeloid progenitor inhibitory factor 1; TNF-α = tumor necrosis factor α; VCAM-1 = vascular cell adhesion molecule 1; VLA-4 = very late activation antigen 4; LFA-1 = lymphocyte function-associated antigen 1 (CD11a); Mac-1 = macrophage-1 receptor; SLe^x = Sialil-Lewis X, CD15s;, CD36 = cluster of differentiation 36; CCR2 = C-C chemokine receptor type 2; TL = T-lymphocytes; MW = muscadine wine; MJ = muscadine juice; SAT = saturated fatty acid; MUFA = monounsaturated fatty acid; PUFA = polyunsaturated fatty acid; K = potassium; Cl = chloride; CO2 = carbon dioxide; ALP = alkaline phosphatase; Ca = calcium; P = phosphorus; Mg = magnesium; Zn = zinc; N = nitrogen; ISI = insulin sensitivity index; TPP = total phenolic content in plasma; TM_0 = Tail Moment in untreated cells (endogenous DNA strand breaks); TM_{300} = Tail Moment in cells treated with 300 μM H_2O_2 for 20 min (exogenous DNA strand breaks); FMD = flow-mediated dilatation (%); GTNMD = glyceryl trinitrate-mediated dilatation (%); ET-1 = endothelin 1; EtOH = ethanol; TGFβ = transforming growth factor β; TNFα = tumor necrosis factor α; W = wine; DW = dealcoholized wine; BUN = blood urea nitrogen; GGT = gamma-glutamyl transferase; GOT = glutamic oxaloacetic transaminase; GPT = glutamic pyruvic transaminase; CVD = cardiovascular disease.

Table S2. Human acute or short-term studies with dealcoholized red wine.

Subjects	Study design	Subjects' Characteristics	Run-In Before Each Intervention	Washout between Interventions	Meal Characteristics	Data Collection After Intervention	Intervention	Plasma Lipid Metabolism Variables	Plasma glucose metabolism variables	Haemostatic System Variables	Plasma Inflammatory and Other Variables	Blood Pressure and Heart Rate Variables	Urinary Variables	Ref
28 M<br>26.6y	Randomized crossover	Healthy	Overnight fast, 72 h no alcohol, poor phenolic diet	3 days	-	Blood: 0, 1, 2, 4, 6, 22 h<br>SBP, DBP, HR: 0, 2, 4, 6 h<br>Urine: 2–0, 0–2, 2–4, 4–6, 6–12, 12–22 h	1. W 147 mL<br>2. Vodka diluted in lemon-flavored water 147 mL<br>3. DW 147 mL<br>4. Lemon-flavored water 147 ml	OxLDL<br>Lipid peroxides<br>TG, HDL	Glucose	-	CRP	SBP<br>DBP<br>HR	Total OHTyr (DOPET)<br>OHTyr-3-*O*-sulfate<br>OHTyr glucuronides<br>Free OHTyr<br>Total HVAL<br>**Total tyrosol**<br>Tyrosol-4-*O*-glucuronide<br>Free tyrosol<br>DOPAC<br>HVA<br>4HPAA	[107]
RESULTS								W vs. BL, water:<br>↓ OxLDL (22 h)<br>↓ Lipid peroxides (22 h)<br>OxLDL and lipid peroxides were inversely related with 0–6 h urinary OHTyr excretion.<br>Vodka vs. DW, water:<br>↑ TG (2 h)<br>W, vodka vs. DW, water:<br>↑ TG (4, 6 h)<br>W, DW vs. water:<br>↑ HDL (2 h)<br>Changes in HDL cholesterol were directly related with the urinary 0–6 h OHTyr excretion	W, vodka vs. DW, water:<br>↓ Glucose (1, 2 h)		NS	W, vodka vs. water:<br>↑ HR (2, 4 h)	W, DW, vodka vs. water:<br>↑ **Total OHTyr (0–6 h)**<br>↑ OHTyr-3-*O*-sulfate (0–6 h)<br>↑ OHTyr glucuronides (0–6 h)<br>↑ **Total tyrosol (0–6 h)**<br>↑ Tyrosol-4-*O*-glucuronide (0–6 h)<br>↓ DOPAC/DOPET (0–6 h)<br>W, DW vs. vodka:<br>↑ **Total OHTyr (0–6 h)**<br>↑ OHTyr-3-*O*-sulfate (0–6 h)<br>W vs. DW:<br>↑ **Total OHTyr (0–6 h)**<br>↑ OHTyr-3-*O*-sulfate (0–6 h)<br>Vodka vs. water:<br>↑ Total HVAL (0–6 h)<br>W vs. DW, vodka, water:<br>↑ Free tyrosol (0–6 h)<br>W vs. DW, vodka:<br>↑ **Total tyrosol (0–6 h)**<br>↑ Tyrosol-4-*O*-glucuronide (0–6 h)<br>DW vs. vodka, water:<br>↑ DOPAC (0–6 h)<br>W, DW vs. water:<br>↑ HVA (0–6 h)<br>W, DW vs. vodka, water:<br>↑ 4HPAA (0–6 h)<br>DW vs. W, vodka:<br>↑ HVA (0–6 h)<br>DW vs. W:<br>↑ 4HPAA (0–6 h)	
25 M (-1)<br>20–65y	Randomized crossover	Healthy, regular drinkers, nonsmokers	Overnight fast, 48 h no alcohol, 2 weeks no dietary supplements	2 weeks, usual drinking, dietary and exercise habits	Light meal	Blood: 0, 2, 4, 24 h<br>BP, HR: every 20 min while awake, every 30 min while asleep, for 24 h	1. RW 375 mL, 41 g alcohol<br>2. DRW 375 mL<br>3. Water 375 ml	-	-	-	20-HETE<br>EETs<br>DHETs<br>ET-1<br>F_2-isoprostanes	SBP<br>DBP<br>HR	-	[108]
RESULTS											W vs. RW, water:<br>↑ 20-HETE (4 h period, 24 h period)	RW vs. DRW, water:<br>↓ SBP (0–4 h and 24 h period)<br>↑ SBP (20–24 h)<br>↓ DBP (0–4 h and 24 h period)<br>↑ HR (0–4 h and 24 h period)		
5 M<br>30–54y	Randomized crossover	Healthy, moderate alcohol consumers, nonsmokers	2 days, the day before all participants consumed the same diet, no alcohol	2 days, usual dietary and lifestyle habits	50g fat overload (FO)	Blood: 0, 3 h	1. Fat overload (FO) + RW 272 mL<br>2. FO + DRW 272 mL<br>3. FO + Gin 100 mL<br>4. FO + No intervention	Chylomicron-LPS, TG, ΔTG, TC, HDL, LDL, ApoA-I, ApoB	Glucose<br>HOMA-IR	-	LPS<br>LBP<br>CRP<br>GGT<br>GPT	-	-	[80]
RESULTS								NS<br><br>Postprandial chylomicron LPS correlated positively with the change in TG between BL and postprandial	NS		NS			
19 M<br>35–68y	Randomized crossover	Healthy, nonsmokers, regular alcohol consumers	3 h no eating or drinking	4 weeks daily intake of the other intervention	Standardized	Blood: 0, 1 h	1. RW 450mL, 41.4 g alcohol<br>2. DRW 450 ml	-	-	-	TEAC<br>NF-kB	-	-	[80]
RESULTS											RW vs. DRW:<br>↑ TEAC (1 h)<br><br>DRW vs. RW, BL:<br>↑ NF-kB (1 h)			[94]

22 M<br>23 ± 1.8y	Double-blind randomized crossover	Healthy, nonsmokers	Overnight fast, 12 h no caffeine or tobacco, 36 h no alcohol	≥ 2 weeks	-	Blood: 0, 2 h<br>Coronary measurements: 0, 30min	1. RW 8.1 ± 0.9 dl, 1.0 g/kg alcohol<br>2. DRW 8.1 ± 0.9 dl	-	-	-	ET-1	Coronary flow velocity and epicardial diameter,<br>HR	-	[109]
RESULTS											RW, DRW vs. BL:<br>↓ ET-1 (2 h)	RW vs. BL:<br>↑ HR		
22 M<br>23 ± 1.8y	Randomized crossover	Healthy, nonsmokers	Overnight fast	≥ 1 week	-	Blood: 0, 1–2 h	1. RW 8.1 ± 0.9 dl, 1.0 g/kg alcohol<br>2. DRW 8.1 ± 0.9 dl<br>3. Cognac 2.4 ± 0.4dl, 1.0 g/kg alcohol	Adiponectin	-	tPAI-1	sE-Selectin, sVCAM-1, sICAM-1, MMP-9, MPO, hsCRP	-	-	[110]
RESULTS								NS		RW vs. BL, DRW, cognac: ↑ tPAI-1 (1–2 h)	Cognac vs. BL: ↓ MMP-9 (1–2 h)			
27	Randomized	Healthy, nonsmokers	Overnight fast, 24 h no alcohol or polyphenol-rich food	-	A polyphenol-poor breakfast was offered 60 min after bolus ingestion.	Blood: 0, 1.5, 6 h	9 subjects:<br>RW 200 mL<br>9 subjects:<br>DRW 175 mL<br>9 subjects:<br>Water 200 ml	-	-	-	Apoptotic T cells, phagocytic activity of granulocytes and monocytes, respiratory burst of granulocytes and monocytes	-	-	[97]
RESULTS											DRW, water vs. BL, RW:<br>↓ T cell apoptosis (6 h)<br><br>Water vs. BL:<br>↓ T cell apoptosis (90 min)<br><br>RW, DRW vs. BL:<br>↑ Monocytes' respiratory burst (90 min, 6 h)<br><br>DRW vs. water:<br>↑ Monocytes' respiratory burst (6 h)			
22 M<br>23 ± 1.8y	Randomized crossover	Healthy, nonsmokers	Overnight fast, 12 h no caffeine or tobacco, 36 h no alcohol	≥ 1 week	Adenosine infusion	CFR: 0, 30 min after each dose<br>BP, HR: 0 h and during the adenosine infusion	1. RW 8.1 ± 0.9 dl administered in 2 doses of 4.0 ± 0.4 dl each<br>2. DRW 8.1 ± 0.9 dl administered in 2 doses of 4.0 ± 0.4 dl each<br>3. Control, no intervention	-	-	-	Plasma antioxidant capacity (ImAnOx assay)	Coronary flow velocity reserve (CFR),<br>Rate pressure product	-	[111]
RESULTS											RW vs. BL:<br>↑ Plasma antioxidant capacity	RW vs. BL:<br>↑ CFR (1 dose RW)		
9 M<br>25–40y	Randomized crossover	Healthy, nonsmokers	Overnight fast, 24 h no alcohol, exercise, fruits, vegetables, dietary supplements, tea, caffeine- or theobromine-containing foods	1 week	The subjects remained in the supine position and abstained from any food or additional beverages during the three-hour study.<br><br>4 subjects, who were assigned to RW, were also treated with uricase	Blood: 0, 0.5, 1, 1.5, 2, 3 h<br>Blood of subjects treated with uricase:<br>0, 1 h	1. RW 3 mL/kg body weight, 195–280 mL total<br>2. DRW 3 mL/kg<br>3. Polyphenols-stripped red wine (PSRW) 3 mL/kg<br>4. Ethanol–water solution (ET) 3 mL/kg<br>5. Water 3 mL/kg	-	-	-	Urate<br>Catechin<br>FRAP	-	-	[112]
RESULTS											RW vs. DRW, PSRW, ET, water:<br>↑ FRAP (1, 1.5, 2, 3 h)<br>Water, ET vs. RW, DRW, PSRW:<br>↓ FRAP (0.5, 1, 1.5, 2 h)<br>ET vs. RW, DRW, PSRW, water:<br>↓ FRAP (3 h)<br>RW, DRW vs. PSRW, ET, water:<br>↑ Catechin (0.5, 1, 1.5, 2, 3 h)<br>RW, PSRW vs. DRW, ET, water:<br>↑ Urate (0.5, 1, 1.5, 2, 3 h)<br>RW vs. PSRW:<br>↑ Urate (1, 1.5, 2 h)<br>DRW vs. RW, PSRW, ET, water:<br>↓ Urate (1.5, 2, 3 h)			

											From the experiments with uricase, it was found that urate accounted for approximately 60% of total plasma antioxidant capacity.			
20:<br>12 M<br>8 W	Double-blind crossover	Healthy, smokers	12 h fast and abstinence from smoking	Usual diet and same meals the day before each study day	1 slice of white bread (30 gr) and 30 gr of cottage-cheese (4% fat)	FMD: 0, 0.5, 1, 1.5 h	1. 1 cigarette (1Cig)<br>2. 1Cig + RW 250 mL<br>3. 1Cig + DRW 250 ml					Artery Diameter, Flow at Rest, Flow in Hyperemia, HR, Flow mediated dilatation (FMD)		[113]
RESULTS												1Cig vs. BL:<br>↓ FMD (0.5, 1 h)<br>↑ HR (0.5 h)<br>1Cig + RW vs. BL:<br>↑ Artery diameter (0.5 h)<br>Overall FMD response after 1Cig was significantly different from 1Cig + RW and 1Cig + DRW		
9 M<br>25–40y	Randomized crossover	Healthy, nonsmokers	Overnight fast, 24 h no alcohol, exercise, fruits, vegetables, dietary supplements, tea, caffeine- or theobromine-containing foods	1 week	The subjects remained in the supine position and abstained from any food or beverages during the 2-h study.	Blood: 0, 0.5, 1, 2 h<br>FMD: 0, 1 h<br>HR, BP: 0, 0.5, 1 h	1. RW 3 mL/kg body weight<br>2. DRW 3 mL/kg<br>3. PSRW 3 mL/kg<br>4. ET 3 mL/kg<br>5. Water 3 mL/kg	-	-	-	Urate<br>Catechin	Brachial artery diameter,<br>FMD,<br>Normalized FMD,<br>HR, BP	-	[114]
RESULTS											RW, DRW vs. PSRW, ET, water:<br>↑ Catechin (0.5, 1, 2 h)<br>RW, PSRW vs. DRW, ET, water:<br>↑ Urate (0.5, 1, 2 h)<br>RW vs. PSRW:<br>↑ Urate (1 h)<br>DRW vs. RW, PSRW, ET, water:<br>↓ Urate (2 h)	RW, PSRW, ET vs. BL:<br>↑ Brachial artery diameter<br>RW vs. BL:<br>↑ Normalized FMD		
27:<br>6 M<br>21 W<br>18–50y	Randomized	Healthy, nonsmokers	Overnight fast, 24 h no alcohol or polyphenol-rich food	-	1 h after the first blood sampling, participants were offered a low in polyphenols breakfast<br><br>Participants were allowed to have lunch between the 1.5 h and the 6 h blood sampling, no alcohol or polyphenol-rich food	Blood: 0, 1.5, 6 h	n = 9<br>RW 200 mL<br>n = 9<br>DRW 175mL<br>n = 9<br>Water 200 ml	-	-	-	TPP, TEAC, Vit.C, Uric acid, Albumin, Bilirubin, Endogenous DNA strand breaks in peripheral leukocytes (TM_0), Exogenous DNA strand breaks in peripheral leukocytes (TM_{300})	-	-	[99]
RESULTS											RW, DRW, water vs. BL:<br>↑ TPP (6 h)<br>RW, DRW vs. BL, water:<br>↑ TPP (1.5 h)<br>RW vs. BL:<br>↓ Vit.C (6 h)<br>↑ Uric acid (1.5 h)<br>DRW vs. BL:<br>↑ Vit.C (6 h)<br>RW, DRW vs. BL:<br>↓ Bilirubin (6 h)<br>↑ TM_0<br>DRW vs. RW, BL, water:<br>↓ TM_{300}<br>Water vs. BL:<br>↑ Albumin (1.5 h)<br>↑ Bilirubin (1.5 h)			
15 M<br>52.4 ± 9.7y	Double-blind randomized crossover	Coronary artery disease, moderate alcohol consumers	12 h overnight fast and absence from smoking, 24 h no vasoactive medication	24 h	-	BP, arterial stiffness measurements: 0, 0.5, 1, 1.5 h	1. RW 250 mL<br>2. DRW 250 ml	-	-	-	-	Augmentation index (AIx),<br>Augmentation index normalized for 75 bpm HR (AI 75),<br>Mean BP,<br>Peripheral SBP,<br>Peripheral DBP,<br>Peripheral PP (pulse pressure),<br>Central SBP,	-	[115]

												Central DBP,<br>Central PP,<br>Arrival time of reflected waves at the central aorta (Δt),<br>HR		
RESULTS												RW, DRW vs. BL:<br>↓ Central SBP<br>↓ AI 75 (0.5, 1 h)<br>↑ HR<br><br>DRW vs. BL:<br>↓ Peripheral DBP<br>↓ Central DBP<br><br>RW vs. DRW:<br>↓ Maximal central SBP<br>↓ Maximal central PP<br>↓ Maximal peripheral PP<br>↓ Maximal AI 75<br><br>RW vs. BL:<br>↓ Central PP<br>↓ AI 75 (1.5 h)		
8 W	Randomized crossover	Postmenopausal, dyslipidemic	-	2 weeks	Mixed meal	Blood: 0, 1, 2, 3, 4, 5, 6 h	1. RW<br>2. DRW<br>3. Water	TC, TG, LDL, HDL, ApoB48	Insulin	-	-	-	-	[116]
RESULTS								DRW vs. water:<br>↓ ApoB48	NS					
16:<br>8 M<br>8 W<br>28.9 ± 6.5y	Double-blind Randomized crossover	Healthy, nonsmokers	1st day: 12 h fast	24 h, usual diet	2nd, 3rd day:<br>1 slice of white bread (30 g) and 30 g of cottage cheese (4% fat)	FMD: 0, 0.25, 0.5, 1, 1.5 h	1st day: 1 cigarette (1Cig)<br>2nd and 3rd day:<br>1. 1 Cig + RW 250 mL<br>2. 1 Cig + DRW 250 ml	-	-	-	-	Brachial artery: vessel size, flow at rest, hyperemia, flow mediated dilatation (FMD)	-	[117]
RESULTS												1 Cig vs. BL:<br>↓ FMD (0.25, 0.5, 1 h)<br><br>1 Cig vs. 1 Cig + RW, 1 Cig + DRW:<br>↓ FMD<br><br>1Cig + RW vs. BL:<br>↑ Vessel size (0.5, 1.5 h)		
6 M<br>31 ± 1.6y	Randomized crossover	Healthy, nonsmokers, moderate alcohol consumers	Overnight fast	1 week, usual diet, no polyphenol- or anthocyanin- rich foods	2 white rolls (150 g), subsequent meals were provided to all subjects for 24 h	Blood: 0, 1, 3, 24 h	1. RW 500 mL<br>2. DRW 500 mL<br>3. 12% ethanol dilution 500 mL<br>4. Red grape juice (RGJ)	-	-	-	TNFα, IL-2, IL-4,<br>Phagocytic activity of neutrophils,<br>Phagocytic activity of monocytes,<br>Phagocytic intensity of neutrophils,<br>Phagocytic intensity of monocytes, Lymphocyte proliferation,<br>NK cell activity	-	-	[118]
RESULTS											A significant time-effect was observed for TNFα, IL-4, phagocytic activity of neutrophils and lytic activity of NK cells independent of the type of beverage consumed.			
6 M<br>Mean 31y	Randomized crossover	Healthy, nonsmokers	Overnight fast	1 week, usual diet, no polyphenol- or anthocyanin- rich foods	2 white rolls (150 g), 200 min after intervention, subjects consumed a complete meal	Blood: 0, 0.33, 0.66, 1, 1.5, 2, 3, 6, 24 h<br>Urine: 0–3, 4–6 h	1. RW 500 mL, 68 mg M-3-G<br>2. DRW 500 mL, 58 mg M-3-G<br>3. RGJ 500mL, 117 mg M-3-G<br>4. 12% ethanol dilution 500 ml	-	Glucose	-	Malvidin-3-glucoside (M-3-G),<br>Malvidin,<br>GGT	-	Malvidin-3-glucoside (M-3-G),<br>Malvidin,<br>M-3-G glucuronates/sulfates (searched in the urine of 2 subjects after RGJ)	[119]
RESULTS									(Sugar content of the beverages were RW 1 g/L, DRW 1 g/L, RGJ 206 g/L)<br>Serum glucose after RGJ peaked at t = 20 min, whereas RW and DRW at t = 60 min		The ingested amount of M-3-G from each beverage shows a linear relationship with the AUC of plasma M-3-G concentrations.<br>Plasma M-3-G and the respective AUC after RGJ was about 2-fold higher than after RW ($p < 0.01$) or DRW ($p < 0.01$)<br>Mean time of individual C_{max} for plasma M-3-G after RGJ consumption was delayed (t = 120 min) as compared to		The urine samples showed the highest concentrations of M-3-G in the samples collected during the first 3 h.<br><br>During the first 6 h, 16 μg M-3-G were excreted, which is less than 0.03 % of the ingested amount.<br><br>A linear relationship was found for the total excretion of M-3-G and the dose ingested (r = 0.386).	

											red wine (t = 50 min, p = 0.008) and dealcoholized red wine (t = 90, p = 0.10).		24 h after interventions, M-3-G was not detected in the urine samples.	
9:<br>5 M<br>4 W	Randomized crossover	Healthy, nonsmokers	14 h fast, 3 days no alcohol, fruit, vegetables, wine, tea, chocolate or coffee	2 days	The subjects were fed a low-flavonoid lunch after collection of the 3 h blood sample	Blood: 0, 0.5, 1, 2, 3, 4, 8 h	1. DRW 120 mL, ~35 mg free catechin<br>2. ARW 120 mL, ~35 mg free catechin	-	-	-	(+)-catechin:<br>8 h AUC<br>t_{max}<br>c_{max}<br>$t_{1/2}$<br>Differences in mean values of these variables were also studied between the two sexes.	-	-	[120]
RESULTS											Plasma (+)-catechin concentrations increased after consumption of a single serving of wine<br>ARW vs. DRW:<br>↓ $t_{1/2}$			
12 M<br>40–63y	Randomized crossover	Healthy, nonsmokers, light-to-moderate drinkers	12 h fast, 24 h no coffee or tea, 2 days no alcohol	1 week, no grape products or any other high phenol-containing foods or drinks	2 plain, low-fat bagels	Blood: 0, 1, 2, 4 h	9 subjects tested all 4 interventions.<br>3 subjects didn't test water.<br>1. RW 5 mL/kg body wt<br>2. PSRW 5 mL/kg body wt<br>3. DRW 5 mL/kg body wt<br>4. Water 5 mL/kg body wt	Ex vivo Cu^{2+}-induced LDL oxidation	-	-	Caffeic acid<br>Protocatechuic acid<br>4-*O*-methylgallic acid<br>Uric acid<br>Ex vivo Cu^{2+}-induced serum oxidation	-	-	[121]
RESULTS								NS			RW, DRW vs. PSRW, water:<br>↑ Caffeic acid<br>↑ 4-*O*-methylgallic acid<br>RW, DRW, PSRW vs. water:<br>↑ Uric acid			
12:<br>8 M<br>4 W<br><40y	Randomized crossover	Healthy, nonsmokers	-	<1 week	Standardized light lunch	Blood: 0, 0.5, 1 h<br>Brachial artery measurements: 0, 0.5, 1 h	1. RW 250 mL<br>2. DRW 250 ml	-	-	-	-	Brachial artery:<br>Resting vessel diameter,<br>Resting blood flow<br>Brachial artery during flow-mediated dilatation examination:<br>Peak blood flow,<br>Blood flow increase,<br>Flow-mediated dilatation (FMD)<br>HR	-	[122]
RESULTS												RW vs. BL, DRW:<br>↑ Vessel diameter<br>↑ Blood flow<br>RW vs. BL:<br>↑ HR<br>RW, DRW vs. BL:<br>↑ Peak blood flow<br>DRW vs. RW:<br>↑ Blood flow increase<br>DRW vs. BL, RW:<br>↑ FMD		
9:<br>5 M<br>4 W<br>29 ± 3y	Randomized crossover	-	14 h fast, 2 days no fruits, vegetables, chocolate, coffee or tea	>2 days	-	Blood: 0, 0.5, 1, 2, 3, 4, 8 h	1. RW 120 mL, ~34 mg free catechin<br>2. DRW 120 mL, ~35 mg free catechin	-	-	-	Total catechin,<br>Methylated catechin metabolites, Unmethylated catechin metabolites,<br>Sulfated catechin metabolites and their relative:<br>half-lives of absorption ($A_{1/2}$)<br>half-lives of elimination ($E_{1/2}$)<br>areas under the curves (AUC)<br>maximum concentrations (C_{max}) maximum times (T_{max})	-	-	[123]
RESULTS											At BL, total catechin was <2 nmol/L.<br>At 1 h, maximum levels of total catechin varied from 50 to 176 nmol/L (RW) and from 46 to 139 nmol/L (DRW) among individuals.<br>At 1 h, methylated metabolites accounted for 20 ± 2% (RW) and 22 ± 3% (DRW) of total catechin.<br>At 1 h, free catechin and free 3′MC accounted for <2% of total catechin metabolites.<br>At 3–4 h, no free catechin was detected in plasma.<br>At 4 h, methylated metabolites accounted for			

only 14 ± 2% (RW) and 12 ± 1% (DRW) of total catechin.<br>At 8 h, levels of total catechin were <25% of the maximum levels.<br>3′MC was not present in a form containing only a sulfate conjugate.<br>DRW vs. RW:<br>↑ $E_{1/2}$ of total catechin (3, 4, 8 h)<br>↑ $E_{1/2}$ of unmethylated catechin metabolites (3, 4, 8 h)

M = men, W = women; y = years; BL = baseline; SBP = systolic blood pressure; DBP = diastolic blood pressure; BP = blood pressure; HR = heart rate; W = wine; RW = red wine; DW = dealcoholized wine; DRW = dealcoholized red wine; OxLDL = oxidized low density lipoproteins; TG = triglycerides; HDL = high density lipoproteins; CRP = c-reactive protein; OHTyr = hydroxytyrosol; HVAL = homovanillyl alcohol; DOPAC = 3,4-dihydroxyphenylacetic acid; HVA = homovanillic acid; 4HPAA = 4-hydroxyphenylacetic acid; 20-HETE = 20-hydroxy eicosatrienoic acid; EETs = epoxyeicosatrienoic acids; DHETs = dihyroxyeicosatetraenoic acid metabolites; ET-1 = endothelin-1; FO = fat overload; LPS = lipopolysaccharide; ΔTG = difference between postprandial and baseline triglycerides' values; TC = total cholesterol; LDL = low density lipoproteins; ApoA-I = apolipoprotein A1; ApoB = apolipoprotein B; HOMA-IR = homeostatic model assessment for insulin resistance; LBP = lipopolysaccharide binding protein; GGT = gamma-glutamyl transferase; GPT = glutamic pyruvic transaminase; TEAC = trolox equivalent antioxidant capacity; NF-kB = nuclear factor kappa-light-chain-enhancer of activated B cells; NS = non significant; tPAI-1 = tissue plasminogen activator inhibitor-1; sE-selectin = soluble E-selectin; sVCAM-1 = soluble vascular cell adhesion molecule 1; sICAM-1 = soluble intercellular adhesion molecule 1; MMP-9 = matrix metalloproteinase 9; MPO = myeloperoxidase; hsCRP = high-sensitivity C-reactive protein; CFR = coronary flow velocity reserve; Rate pressure product = systolic blood pressure x heart rate; FRAP = ferric reducing antioxidant power; min = minutes; h = hour; PSRW = polyphenols-stripped red wine; ET = ethanol-water solution; FMD = flow mediated dilatation; TPP = total phenolic content in plasma; TM_0 = Tail Moment in untreated cells (endogenous DNA strand breaks); TM_{300} = Tail Moment in cells treated with 300 μM H_2O_2 for 20 min (exogenous DNA strand breaks); 1Cig = 1 cigarette; TNFα = tumor necrosis factor alpha; IL = interleukin; NK cell = natural killer cell; RGJ = red grape juice; GGT = γ-glutamyl-transferase; ARW = alcoholized red wine; 8 h AUC = area under curve at 8 h (of plasma concentration); t_{max} = time to maximum plasma concentration; c_{max} = maximum plasma concentration; $t_{1/2}$ = elimination half-life.
